# To the Point

**Published:** 1872-04

**Authors:** 


### The Bistoury.

ELMIRA, N. Y., APRIL, 1872.

The BISTOURY is published Quarterly, upon the 1st of April, July October, and January, at Fifty Cents a year, in advance.

### TO THE POINT.

THE BISTOURY makes its eighth annual bow to the public, and assures them that it is in a prosperous condition, and as jolly as usual. It rather enjoys appearing before them four times yearly, freighted with such wholesome advice, and is glad to know that its visits are appreciated, as is evinced by the large number of subscriptions received since its last issue. BISTOURY has grown so largely, that like most prosperous people, it has assumed a sort of independent attitude, and is somewhat inclined to be saucy.—It therefore desires to inform those persons—and they are numerous — who have received it for the past three years, without paying for it, that they *must* discharge their obligations to us, *pay up and quit!* We have quite enough good, intelligent, and paying subscribers, without them, therefore inclose the delinquents, the necessary blanks and envelopes in this number, so that they can discharge their indebtedness, and order the journal discontinued, if they so desire. We publish the Newspaper law, in the Chit Chat, for their benefit, in which they will discover that a journal cannot be discontinued until all arrearages are paid. We will therefore pay no attention to "orders to discontinue," unless accompanied with the *cash*. Those who have taken the Bistoury for three years, without once paying for it, owe $1.50; and that is just the sum we require, so please oblige us with it, and we will "play quits" in future. Otherwise we will continue to send the journal to delinquents until the sum amounts to sufficient to warrant us in sending out agents, compelling payment of those who have not died of mortification — by reason of their meanness. One dollar and fifty cents is a small sum to be paid by *one* man, but when a thousand owe that amount to another—if our arithmetic serves us rightly—it amounts to the handsome sum of $1,500; and the more we think about it, the more we are determined not to lose it,—would you?

One promise, made in our last issue, we must ask our friends to excuse us from fulfilling, for the reason that so many of our subscribers have written us *not* to page the Bistoury as we there proposed. So, we will page it in regular order, as before, making a handsome volume, brim full of sound, medical advice, every two or three years.

Thanking our friends, all over this land, for their kind exertions in extending the circulation of our journal, we enter upon another editorial year, with the consciousness of doing good, and with a determination to merit approbation.

